# The complete mitogenome of the invasive Japanese mud snail *Batillaria attramentaria* (Gastropoda: Batillariidae) from Elkhorn Slough, California, USA

**DOI:** 10.1080/23802359.2019.1688719

**Published:** 2019-11-13

**Authors:** Hartnell College Genomics Group, Paulina Andrade, Lisbeth Arreola, Melissa Belnas, Estefania Bland, Araceli Castillo, Omar Cisneros, Valentin Contreras, Celeste Diaz, Kevin T. Do, Carlos Donate, Estevan Espinoza, Nathan Frater, Garry G. Gabriel, Eric A. Gomez, Gino F. Gonzalez, Myrka Gonzalez, Paola Guido, Dylan Guidotti, Mishell Guzman Espinoza, Ivan Haro, Javier Hernandez Lopez, Caden E. Hernandez, Karina Hernandez, Jazmin A. Hernandez-Salazar, Jeffery R. Hughey, Héctor Jácome-Sáenz, Luis A. Jimenez, Eli R. Kallison, Mylisa S. King, Luis J. Lazaro, Feifei Zhai Lorenzo, Isaac Madrigal, Savannah Madruga, Adrian J. Maldonado, Alexander M. Medina, Marcela Mendez-Molina, Ali Mendez, David Murillo Martinez, David Orozco, Juan Orozco, Ulises Ortiz, Jennifer M. Pantoja, Alejandra N. Ponce, Angel R. Ramirez, Israel Rangel, Eliza Rojas, Adriana Roque, Beatriz Rosas, Colt Rubbo, Justin A. Saldana, Elian Sanchez, Alicia Steinhardt, Maria O. Taveras Dina, Judith Torres, Silvestre Valdez-Mata, Valeria Vargas, Paola Vazquez, Michelle M. Vazquez, Irene Vidales, Frances L. Wong, Christian S. Zagal, Santiago Zamora, Jesus Zepeda Amador

**Affiliations:** Division of Mathematics, Science, and Engineering, Hartnell College, Salinas, CA, USA

**Keywords:** *Batillaria attramentaria*, Cerithioidea, Elkhorn Slough, invasive species, mitogenome

## Abstract

Genomic analysis of the invasive marine snail *Batillaria attramentaria* from Elkhorn Slough, Moss Landing, California, USA using 150 bp paired-end Illumina sequences resulted in the assembly of its complete mitogenome. The mitogenome is 16,095 bp in length and contains 2 rRNA, 13 protein-coding, and 22 tRNA genes (GenBank Accession MN557850). Gene content and organization of *B. attramentaria* are identical to the Turritellidae and Pachychilidae. The phylogenetic analysis of *B. attramentaria* resolves it in a fully supported clade with these same two families in the superfamily Cerithioidea. Nucleotide BLAST searches of the Elkhorn Slough *cox1* gene of *B. attramentaria* yielded identical sequences from invasive populations from California and British Columbia, and native populations from northeastern and central Japan. These data show that mitogenome sequencing is a useful tool for studying the classification and phylogenetic history Cerithioidea.

The Batillaridae are aquatic snails that occur along coastal intertidal and shallow estuarine lagoons. The family contains four genera *sensu stricto* and 14 species (Ozawa et al. 2009). One of these genera, *Batillaria*, includes a highly invasive marine snail *B. attramentaria* (Byers 1999, 2000; Miura et al. 2006). *Batillaria* was first introduced in March of 1930 into Elkhorn Slough, California, USA in a shipment of 150 boxes of oysters that arrived from Japan (Bonnot 1935). This shipment and perhaps others that followed inadvertently introduced *B. attramentaria* and more than 50 other invertebrates into the slough (Wasson et al. 2001). The population of *B. attramentaria* in Elkhorn Slough was estimated at 1 billion, and in California, it was responsible for displacing the native snail *Cerithidea californica* (Byers 1999). Here, we performed high-throughput sequencing on a specimen of *B. attramentaria* from Elkhorn Slough to determine its mitogenome structure and phylogenomic relationship to other gastropods in the superfamily Cerithioidea.

DNA was extracted from *B. attramentaria* (Voucher Specimen-Hartnell College #263) following the protocol of Lindstrom et al. (2011). The 150 bp PE Illumina library construction and sequencing was performed by myGenomics, LLC (Alpharetta, Georgia, USA). The mitogenome was assembled using 22,355,422 reads with the default de novo settings in MEGAHIT (Li et al. 2015). The genes were annotated with NCBI ORFfinder and MITOS (Bernt et al. 2013). Alignment of the *B. attramentaria* mitogenome to other Gastropoda was completed with MAFFT (Katoh and Standley 2013). The RAxML analysis was run using T-REX (Boc et al. 2012) with the GTR + gamma model and 1000 bootstraps. The tree was visualized with TreeDyn 198.3 at Phylogeny.fr (Dereeper et al. 2008).

The mitogenome of *B. attramentaria* (GenBank MN557850) is 16,095 bp in length and has a base composition of 30.14% A, 35.24% T, 17.01% G, and 17.61% C. It contains 2 rRNA (rnl, rns), 13 electron transport and oxidative phosphorylation genes, and 22 tRNAs (*tRNA-Leu* and *tRNA-Ser* are duplicated). All 13 protein-coding genes initiate with the ATG codon. Most of the genes terminate with the TAA codon, however, *cox2*, *nad1*, and *nad2* terminate with TAG. The mitogenome of *B. attramentaria* is identical in gene content and organization to *Turritella* (Family Turritellidae) and *Tylomelania* (Pachychilidae) (Hilgers et al. 2016; Zeng et al. 2016). These three families have tandemly arranged *tRNA-Arg* and *tRNA-Gln* genes. In other published Cerithioidea mitogenomes, *tRNA-Gln* is situated on the chromosome with *tRNA-Ser*, and *tRNA-Arg* is positioned in between *tRNA-Cys* and *tRNA-Ala*. Phylogenetic analysis of *B. attramentaria* resolves it in a fully supported clade with *Turritella* and *Tylomelania* in the superfamily Cerithioidea (Figure 1). Strong et al. (2011) previously reported a similar phylogenetic relationship between the Batillariidae, Turritellidae, and Pachychilidae based on 16S and 28S rRNA DNA sequences. Analysis of the *B. attramentaria cox1* sequence found identical sequences from invasive specimens from Elkhorn Slough, California and British Columbia, Canada, and native specimens from northeastern and central Japan (Kojima et al. 2004; Miura et al. 2006; Castelin et al. 2016).

**Figure 1.**
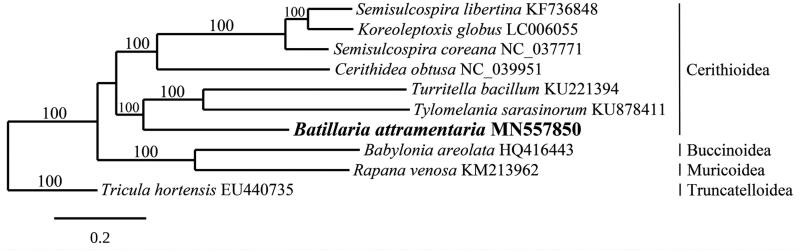
Maximum-likelihood phylogram of *Batillaria attramentaria* and related gastropod mitogenomes. Superfamilies are listed to the right. Numbers along branches are bootstrap supports based on 1000 nreps. The legend below represents the scale for nucleotide substitutions.
